# The Environmental and Socioeconomic Effects and Prediction of Patients With Tuberculosis in Different Age Groups in Southwest China: A Population-Based Study

**DOI:** 10.2196/40659

**Published:** 2023-01-13

**Authors:** Wen Wei, Lan Xia, Jianlin Wu, Zonglei Zhou, Wenqiang Zhang, Rongsheng Luan

**Affiliations:** 1 Department of Epidemiology and Biostatistics West China School of Public Health and West China Fourth Hospital Sichuan University Chengdu China; 2 Department of Tuberculosis, Center for Disease Control and Prevention of Sichuan Province Chengdu China

**Keywords:** tuberculosis, risk factors, age, sex, prediction, TB control, tuberculosis control

## Abstract

**Background:**

While the End Tuberculosis (TB) Strategy has been implemented worldwide, the cause of the TB epidemic is multifactorial and not fully understood.

**Objective:**

This study aims to investigate the risk factors of TB and incorporate these factors to forecast the incidence of TB infection across different age groups in Sichuan, China.

**Methods:**

Correlation and linear regression analyses were conducted to assess the relationships between TB cases and ecological factors, including environmental, economic, and social factors, in Sichuan Province from 2006 to 2017. The transfer function-noise model was used to forecast trends, considering both time and multifactor effects.

**Results:**

From 2006 to 2017, Sichuan Province had a reported cumulative incidence rate of 1321.08 cases per 100,000 individuals in male patients and 583.04 cases per 100,000 individuals in female patients. There were significant sex differences in the distribution of cases among age groups (trend *χ*^2^_25_=12,544.4; *P*<.001). Ganzi Tibetan Autonomous Prefecture had the highest incidence rates of TB in both male and female patients in Sichuan. Correlation and regression analyses showed that the total illiteracy rate and average pressure at each measuring station (for individuals aged 15-24 years) were risk factors for TB. The protective factors were as follows: the number of families with the minimum living standard guarantee in urban areas, the average wind speed, the number of discharged patients with invasive TB, the number of people with the minimum living standard guarantee in rural areas, the total health expenditure as a percentage of regional gross domestic product, and being a single male individual (for those aged 0-14 years); the number of hospitals and number of health workers in infectious disease hospitals (for individuals aged 25-64 years); and the amount of daily morning and evening exercise, the number of people with the urban minimum living standard guarantee, and being married (for female individuals aged ≥65 years). The transfer function-noise model indicated that the incidence of TB in male patients aged 0-14 and 15-24 years will continue to increase, and the incidence of TB in female patients aged 0-14 and ≥65 years will continue to increase rapidly in Sichuan by 2035.

**Conclusions:**

The End TB Strategy in Sichuan should consider environmental, educational, medical, social, personal, and other conditions, and further substantial efforts are needed especially for male patients aged 0-24 years, female patients aged 0-14 years, and female patients older than 64 years.

## Introduction

Tuberculosis (TB) has been the leading cause of the global disease burden. Approximately 25% of the population worldwide are infected with *M tuberculosis* [1]. In 2019, according to the World Health Organization, there were approximately 10 million new cases of TB globally and 1.2 million deaths [2]. Of note, almost 90% of individuals who are infected with TB each year originate from low-income countries [2]. Poor health services, malnutrition, and crowded working and living conditions in these countries leads to the increased risk of TB across populations. Fighting poverty has become a major theme for the World Health Organization, which aims to end the global TB epidemic using “the End TB Strategy” [2]. According to the End TB Strategy, the targeted percentage reduction in the absolute number of TB deaths and incidence rate for 2035 are 95% and 90% of the 2015 baseline, respectively [1]. However, the cause of the TB epidemic is multifactorial and not fully understood.

Most people (approximately 90%) develop the disease in adulthood, with men being more susceptible than women [1-4]. Previous ecological studies have demonstrated a significant association between TB cases and ecological factors, including environmental, economic, health, and social conditions [3-11]. Although ecological risk factors for TB at the population and individual levels have raised great concerns, these results are not consistent [1,2,11-16]. The cause of TB varies in different populations and countries, especially in different groups (eg, of different ages and sexes) or in areas with a high TB burden [1], which is a major barrier for the End TB Strategy. China had the third-largest TB burden in the world in 2019, while Sichuan Province, known for its geographical and ethnic diversity, has a top-ranked TB burden, thus providing the opportunity to comprehensively identify specific risk factors in a complex background.

Furthermore, elucidating the trend of TB incidence with identified TB factors helps to assess the effectiveness of containing measurements that may aid policy maker decisions and public health practice. Therefore, our research aimed to identify these potential risk factors for TB in Southwest China using multiple regression and to predict the trend of TB incidence using a transfer function-noise (TFN) model [17-21]. The overarching goal of our study was to gain insight into the secular TB trend, providing implications for advancing TB prevention and control strategies to achieve the targets of the End TB Strategy.

## Methods

### Data Sources and Objects

#### Geographic Information

We obtained data from China’s Geographic Information Center on Sichuan Province in 2009. The map of county-level administrative divisions included prefectures, counties, cities, and districts. In total, Sichuan Province has 21 cities or prefectures and 181 districts, counties, and cities, with a total of 1.4 million inhabitants.

#### Social, Economic, Environmental, Education, and Health Information

Data were extracted from the 2006-2017 Statistical Yearbook of Sichuan Provincial Bureau of Statistics. All TB cases were grouped by sex and age. The age groups were as follows: children (aged 0-14 years), youths (aged 15-24 years), adults (aged 25-64 years), and older individuals (aged >64 years).

From the age-stratified population of the “Epidemic Information Network Direct Reporting System” from 2006 to 2017, we enrolled the subpopulation in Sichuan Province. All population data were for permanent residents, specific to county administrative divisions, and included population data by age and sex. Information on TB and HIV/AIDS was obtained from the TB Information Management System of the Chinese Disease Prevention and Control Information System and the Statistical Yearbook published by the Sichuan Provincial Bureau of Statistics. The incidence of TB was analyzed in different age groups.

### Statistical Analysis

The ecological analysis used data such as case reports, registered case data, and ecological information to explore the risk factors related to the prevalence of TB. Data on TB cases and the incidence rate of TB were collected per administrative division, sex, and age group. The variables for each model were chosen by auto-modeling.

The 128 ecological factors were all obtained from 12 years (2006-2017) of data. The transfer function model was fitted by the autoregressive integrated moving average (ARIMA) model by time series [17-21], using expert modeling and adding independent variables for the model fitted to choose the variables through the fitted data and prediction data; we used 3 different models (the Grey model, the ARIMA model, the TFN model) to identify the model that most closely aligned with the real TB data of 2018.

### TFN Model

The expert modeler selected the optimal model from multiple fitted models, when the *R*^2^ value reached the ideal state. The linear regression analysis adopts the stepwise regression analysis method and uses multiple models to fit and to achieve a better *R*^2^ value in four different age groups. Finally, only the best model was used for display. Multiple stepwise regression was carried out for multivariate analysis (stepwise regression rules: F-to-enter≥3.840, F-to-remove≤2.710). The models were established by each factor, stepwise in and out of the models. Multimedia Appendix 1 shows the stepwise regression summary; when the *R*^2^ and standard estimated error reached the best value, the model concluded.

Univariate analyses (Pearson correlation analysis) and multivariate analyses (regression analysis) were used to analyze the protective factors and risk factors for TB in Sichuan Province according to sex and age group (trend *χ*^2^_25_=12,544.4; *P*<.001). Statistical analysis (descriptive analysis and cluster analysis of spatiotemporal scans) and prediction of TB incidence in 2035 were performed according to sex and age.

The autocorrelation test of the residuals uses the Durbin-Watson (DW) test, with the following test statistic:



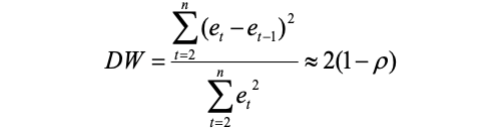



DW values occur in a range from 0 to 4 as follows: a DW value of 0 indicates complete positive autocorrelation, values between 0 and 1.5 indicate positive autocorrelation, values between 1.5 and 2.5 indicate no autocorrelation, values between 2.5 and 4 indicate negative autocorrelation, and a value of 4 indicates complete negative autocorrelation. In addition, the autocorrelation function and partial autocorrelation function show whether the data sequence reached a stable state. At the same time, the *R*^2^ and Bayesian information criterion values of the TFN model were used before and after data unit standardization to find the best model. The closer the DW value is to 2, the more independent the observations of multiple linear regression are.

Considering the effect of time and multiple factors, we used Panel regression, Poisson regression, and Lasso regression for the analysis; however, the data were not suitable for these regressions; for example, our study has a large number of factors to explore the relationship, so the Panel regression could not include all 128 factors and ID and time to fit. The pilot analyses revealed that the random effect model was better than the pool model and fixed effect model. For these reasons, we did not use other regression methods.

We included all reported cases of TB during 2006-2017; these data possibly contain information and selection bias. The TFN model is a multivariate time series analysis method that can be seen as a combination of the ARIMA model and a multiple regression model. We used SPSS 23.0 (IBM Corp) and ArcGis Map 10.6 (ESRI Inc) to create spatiotemporal scans and to conduct the analyses that predicted TB trends by sex and age. The three main steps were as follows: model identification, parameter estimation, and model testing. For the calculation of the *P* value, the analysis was applied under the assumptions of unequal variances and a statistical significance of *P*<.05.

### Ethics Approval

Data collection of TB was required by the Law of the People’s Republic of China on Prevention and Treatment of Infectious Diseases. The ethics approval in this study was granted by the Ethics Committee of Sichuan Center for Disease Control and Prevention (SCCDCIRB2022-001).

## Results

### Annual TB Cases and Incidence Rate

From 2006 to 2017, Sichuan Province reported 548,584 cases of pulmonary TB in male patients, with a reported cumulative incidence rate of 1321.08 cases per 100,000 individuals (average 110.09 cases/100,000 individuals), and 235,149 cases of pulmonary TB in female patients, with a reported cumulative incidence rate of 583.04 cases per 100,000 individuals (average 48.59 cases/100,000 individuals). Thus, there were approximately 2.33 times more cases in male patients than in female patients. The reported cumulative incidence of TB in Sichuan Province from 2006 to 2017 was 961.71 cases per 100,000 individuals (average 80.14 cases/100,000 individuals). These TB cases mainly occurred in individuals aged 15-64 years, which accounted for 82.02% (n=642,808) of the total cases.

**Figure 1:**
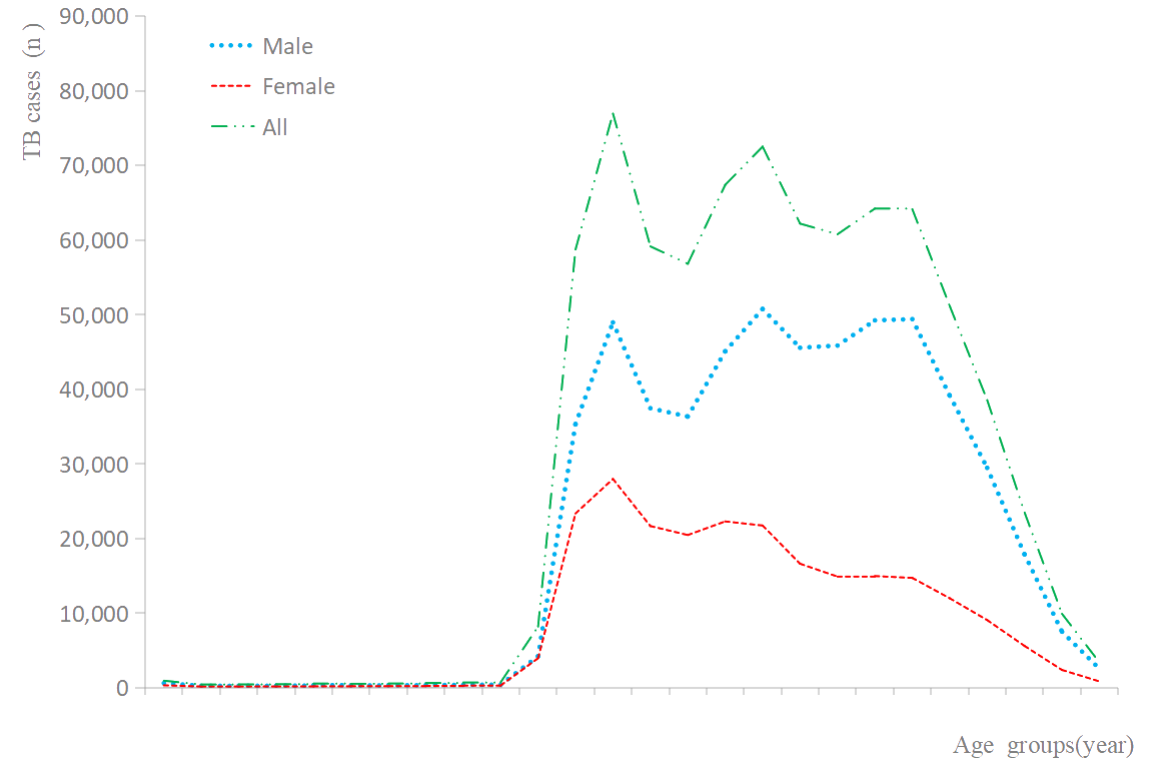
TB cases in Sichuan Province of China during 2006-2017. TB: tuberculosis.

**Figure 2:**
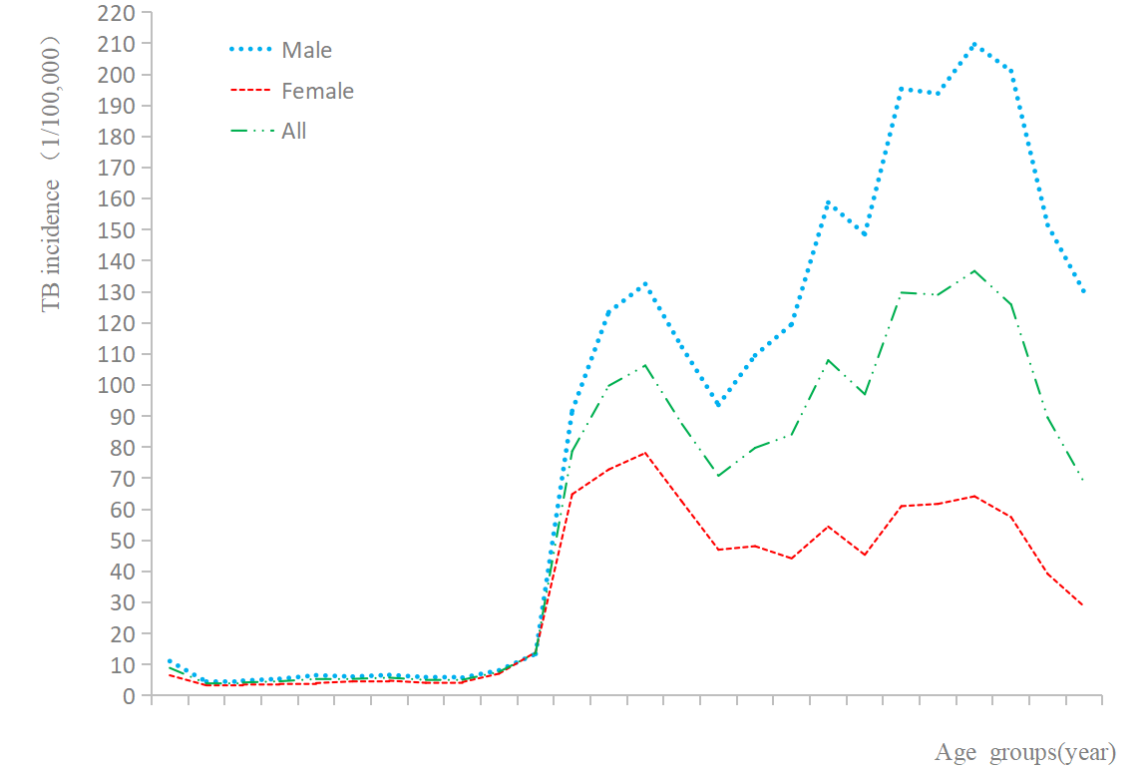
TB incidence rate in Sichuan Province of China during 2006-2017. TB: tuberculosis.

As shown in Figure 1, there were sex differences in the distribution of cases among age groups, and these differences were significant (trend *χ*^2^_25_=12,544.4; *P*<.001). The number of TB cases in Sichuan Province peaked in individuals aged 20-24 years and gradually decreased in individuals older than 64 years (Figure 1). During these 12 years (2006-2017), the incidence rate in those aged 80-85 years was lower than in those aged 60-79 years, while individuals older than 70 years had the highest TB incidence peak (Figure 2).

### Geographic Distribution

**Figure 3:**
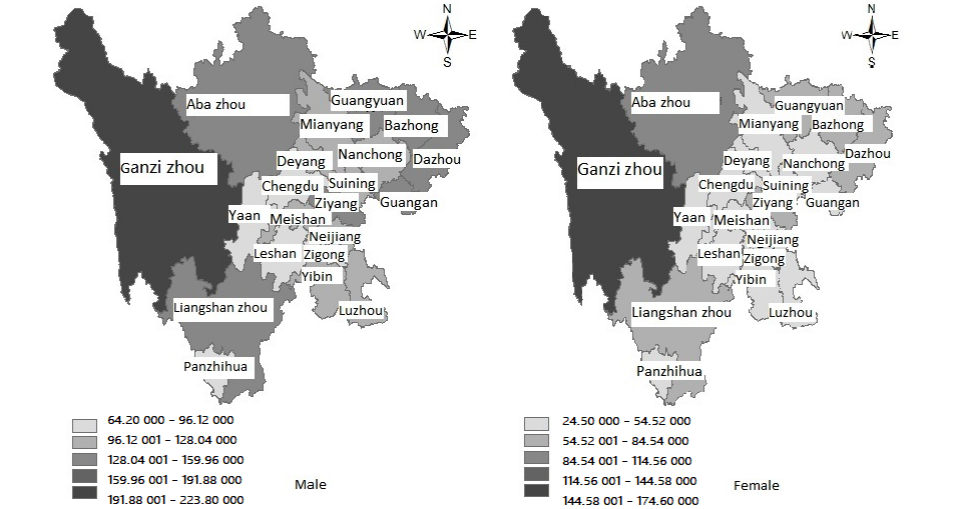
The incidence of male and female TB in Sichuan Province of China during 2006-2017(1/100,000). TB: tuberculosis.

Figure 3 displays the TB cumulative incidence in 21 cities/prefectures during this time period. The 5 cities/prefectures with the highest incidence rates of TB in male patients were Ganzi Tibetan Autonomous Prefecture, Aba Tibetan and Qiang Autonomous Prefecture, Guangyuan City, Liangshan Yi Autonomous Prefecture, and Dazhou. The five cities/prefectures with the lowest incidence rates of TB in male patients were Chengdu, Panzhihua, Ya’an, Leshan, and Zigong. In female patients, the top five cities/prefectures according to TB incidence rates were Ganzi Tibetan Autonomous Prefecture, Aba Tibetan and Qiang Autonomous Prefecture, Liangshan Yi Autonomous Prefecture, Guangyuan City, and Dazhou; the bottom five were Chengdu, Zigong, Ya’an, Leshan, and Panzhihua.

### Relationships Among the Variables

Across the four age groups, there were significant associations between TB incidence and the environmental and socioeconomic effects in five aspects, including other diseases as well as health resources; economic and social factors; pollutant, forestry, and meteorology variables; population structure, marriage, and habit variables; and educational investment and education level (all *P*<.05; Multimedia Appendix 1, Tables S1-S5).

### Multivariate Analysis

As shown in Table 1, a linear regression analysis was performed on the incidence of TB and the various factors, and a multivariate analysis was performed on the four age groups in the whole population, the male population, and the female population. The regression model exhibited multivariate multicollinearity; the DW values for the four age groups (individuals aged 0-14, 15-24, 25-64, or ≥65 years) were 2.433, 1.340, 2.491, and 3.114, respectively.

In addition, in those aged 0-14 years, the factors affecting the risk of TB were as follows: the number of families with the urban minimum living standard guarantee, average wind speed, number of patients with invasive pulmonary TB discharged, number of people with the rural minimum living standard guarantee, total health expenditure as a percentage of regional gross domestic product (GDP), and being a single man. These factors reduced the risk of TB. The incidence of TB in those aged 15-24 years, sex ratio, number of forest fires, and regular exercise rate of adults increased the risk of TB in children (Table 1).

In those aged 15-24 years, the incidence of TB in those aged 0-14 years, total illiteracy rate, number of infectious disease hospitals, enrollment number (secondary school and above), exposure to secondhand smoke among nonsmokers (%), fraction of married men (%), number of forest fires, and number of junior high school boys (%) increased the risk of TB (Table 1).

In those aged 25-64 years, the number of hospitals, number of health workers in infectious disease hospitals, TB rate in those aged ≥65 years, daylight duration, and male life expectancy decreased the risk of TB (Table 1).

Finally, in the group of people older than 64 years, the risk of TB was increased by the following factors: the rate of TB cases in female patients, cumulative precipitation over 24 hours (8 PM to 8 PM), rate of TB in those aged 25-64 years, total health expenditure as a percentage of regional GDP, number of families with the rural minimum living standard guarantee, rate of TB in patients aged 15-24 years, and civil medical assistance. The risk of TB decreased with the amount of daily morning and evening exercise and the number of people with the urban minimum living standard guarantee and in women who were married (Table 1).

**Table 1:** Multivariate analysis of tuberculosis (TB) incidence by age group (individuals aged 0-14, 15-24, 25-64, or ≥65 years) in Sichuan Province.

Model and variables		B (95% CI)	*T* test (*df*)	*P* value
**Model 1 (dependent variable: TB incidence in those aged 0-14 years; Durbin-Watson value: 2.433)**				
	(constant)	–8.259 (–8.269 to –8.249)	–10626.577 (11)	<.001
	TB rate in those aged 15-24 years	1.01E-01 (0.101 to 0.101)	14772.916 (11)	<.001
	Sex ratio	8.387 (8.379 to 8.394)	14578.003 (11)	<.001
	Number of forest fires	0.001 (0.001 to 0.001)	3593.154 (11)	<.001
	Number of families with the minimum living standard guarantee in urban areas (households)	–7.438E-07 (0 to 0)	–2011.933 (11)	<.001
	Average wind speed	–0.005 (–0.005 to –0.005)	–1576.859 (11)	<.001
	Number of patients with invasive TB discharged	–1.505E-05 (0 to 0)	–4259.893 (11)	<.001
	Number of people with the minimum living standard guarantee in rural areas (person)	–1.41E-07 (0 to 0)	–1813.338 (11)	<.001
	Regular exercise rate	1.82 (1.8 to 1.839)	1199.617 (11)	.001
	Total health expenditure as a percentage of regional GDP^a^ (%)	–0.007 (–0.008 to –0.007)	–377.144 (11)	.002
	Unmarried men (%)	–0.001 (–0.001 to –0.001)	–105.253 (11)	.006
**Model 2 (dependent variable: TB incidence in those aged 15-24 years; Durbin-Watson value: 1.340)**				
	(constant)	–2541.142 (–2542.913 to –2539.371)	–18234.07 (11)	<.001
	TB rate in those aged 0-14 years	6.227 (6.226 to 6.228)	82051.414 (11)	<.001
	Total illiteracy rate (%)	1.789 (1.788 to 1.789)	27895.184 (11)	<.001
	Average pressure at each measuring station	0.307 (0.307 to 0.307)	18170.576 (11)	<.001
	Number of infectious disease hospitals	0.854 (0.853 to 0.854)	20744.736 (11)	<.001
	Enrollment number (secondary school and above)	0.000004396 (0 to 0)	13489.017 (11)	<.001
	Exposure to secondhand smoke among nonsmokers (%)	0.094 (0.094 to 0.094)	11336.041 (11)	<.001
	Male illiteracy rate (%)	–0.617 (–0.618 to –0.615)	–5377.636 (11)	<.001
	Married men (%)	0.083 (0.083 to 0.083)	3963.885 (11)	<.001
	Number of forest fires	0.001 (0.001 to 0.001)	895.709 (11)	.001
	Junior high school boys (%)	0.004 (0.004 to 0.005)	128.503 (11)	.005
**Model 3 (dependent variable: TB incidence in those aged 25-64 years; Durbin-Watson value: 2.491)**				
	(constant)	10.268 (10.218 to 10.317)	2642.64 (11)	<.001
	TB rate	1.123 (1.123 to 1.123)	99793.924 (11)	<.001
	Number of hospitals	–0.009 (–0.009 to –0.009)	–54131.67 (11)	<.001
	Number of health workers in infectious disease hospitals	–0.006 (–0.006 to –0.006)	–32021.619 (11)	<.001
	TB rate in those aged >64 years	–0.048 (–0.048 to –0.048)	–26994.198 (11)	<.001
	Daylight duration	–0.178 (–0.178 to –0.178)	–14248.119 (11)	<.001
	Life expectancy	0.421 (0.418 to 0.424)	1887.559 (11)	<.001
	Diabetes mortality rate (1/100,000)	0.117 (0.116 to 0.117)	4809.176 (11)	<.001
	Total expenditure per person in urban residents (¥)	0.00002666 (0 to 0)	915.623 (11)	.001
	Number of public health workers per 1000 people	0.062 (0.06 to 0.065)	356.061 (11)	.002
	Male life expectancy	–0.032 (–0.034 to –0.029)	–171.099 (11)	.004
**Model 4 (dependent variable: TB incidence in those aged >64 years; Durbin-Watson value: 3.114)**				
	(constant)	–28.128 (–28.41 to –27.846)	–1266.037 (11)	.001
	TB rate in women	2.98E+00 (2.979 to 2.98)	70646.596 (11)	<.001
	Amount of daily morning and evening exercise	–5.24E-05 (0 to 0)	–29473.402 (11)	<.001
	Cumulative precipitation in 24 hours (8 PM to 8 PM)	0.985 (0.985 to 0.985)	44529.735 (11)	<.001
	TB rate in those aged 25-64 years	1.391 (1.39 to 1.392)	15700.111 (11)	<.001
	Number of people with the urban minimum living standard guarantee (person)	–0.00001256 (0 to 0)	–3962.971 (11)	<.001
	Total health expenditure as a percentage of regional GDP (%)	1.51 (1.506 to 1.513)	5632.666 (11)	<.001
	Number of families with the rural minimum living standard guarantee (households)	0.000001464 (0 to 0)	1182.888 (11)	.001
	TB rate in women aged 15-24 years	0.132 (0.13 to 0.133)	1161.821 (11)	.001
	Married women (%)	–0.192 (–0.196 to –0.189)	–676.252 (11)	.001
	Civil medical assistance (times used)	1.04E-09 (0 to 0)	44.526 (11)	.01

^a^GDP: gross domestic product.

### Forecast Trend

**Figure 4:**
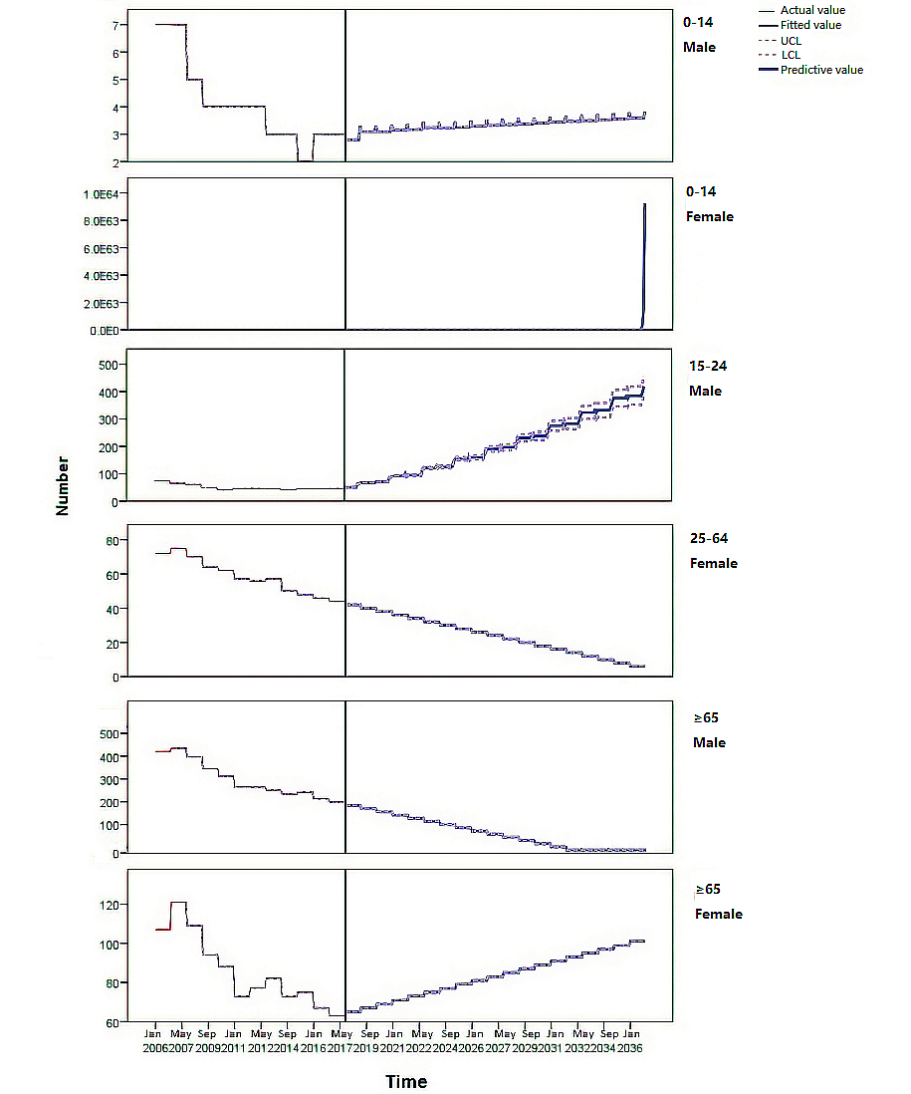
The transfer function-noise model predicts the trend of age-specific incidence rates (male/female: aged 0-14, 15-24, 25-64, and ≥65 years). LCL: lower confidence limit; UCL: upper confidence limit.

In addition, the TFN model predicted that in 2035, the incidence of TB in male patients aged 0-14, 15-24, 25-64, and ≥65 years per 100,000 individuals would be 4.00, 377.08, 0.00, and –54.00, respectively (Figure 4). Our model predicted that the incidence of TB in males aged 0-14, 15-24, 25-64, and ≥65 years would not fall to 0 by 2035 and that the incidence rate of TB in male patients aged 0-14 and 15-24 years would increase.

Moreover, the TFN results showed that the incidence of TB among female patients aged 0-14, 15-24, 25-64, and ≥65 years per 100,000 individuals in 2018 was 3.00, unpredictable, 42.00, and 65.00, respectively. However, in 2035, these rates were predicted to increase, and TB incidence among female patients aged 0-14 years was predicted to be higher than among women aged ≥65 years, which might also exhibit an upward trend (Figure 4).

### Model Selection

The TFN model was used to fit the number and incidence rate of TB cases reported in Sichuan Province during 2006-2017, establish and judge the models, and predict TB incidence in Sichuan Province during 2018-2035. The TFN model was fitted for different groups to determine the TB incidence. The *R*^2^ and Bayesian information criterion of the models and evaluations were all ideal, as shown in Table S6 in Multimedia Appendix 1.

The TFN models used female and male data separately in the multivariate models, and the total of all cases was 783,735. From 2006 to 2017, Sichuan Province reported 548,585 cases of pulmonary TB in male patients, with a reported incidence rate of 11 cases per 100,000 individuals, and 235,150 cases of pulmonary TB in female patients, with a reported cumulative incidence rate of 4.85 cases per 100,000 individuals. There were 12-year observations per model at the population level. For each model, using stepwise regression methods, 10 well-fitting models were established (a better *R*^2^ value), and the rule was carried out for multivariate analysis (stepwise regression rules: F-to-enter≥3.840, F-to-remove≤2.710).

Additionally, we adjusted the variables of the data to be standardizing variables, and the result did not change. The findings also show that the data trend is relatively stable after multiple data processing, such as splitting or logarithmic processing. Both the destandardized and standardized results and indicators showed that the models were the same.

**Figure 5:**
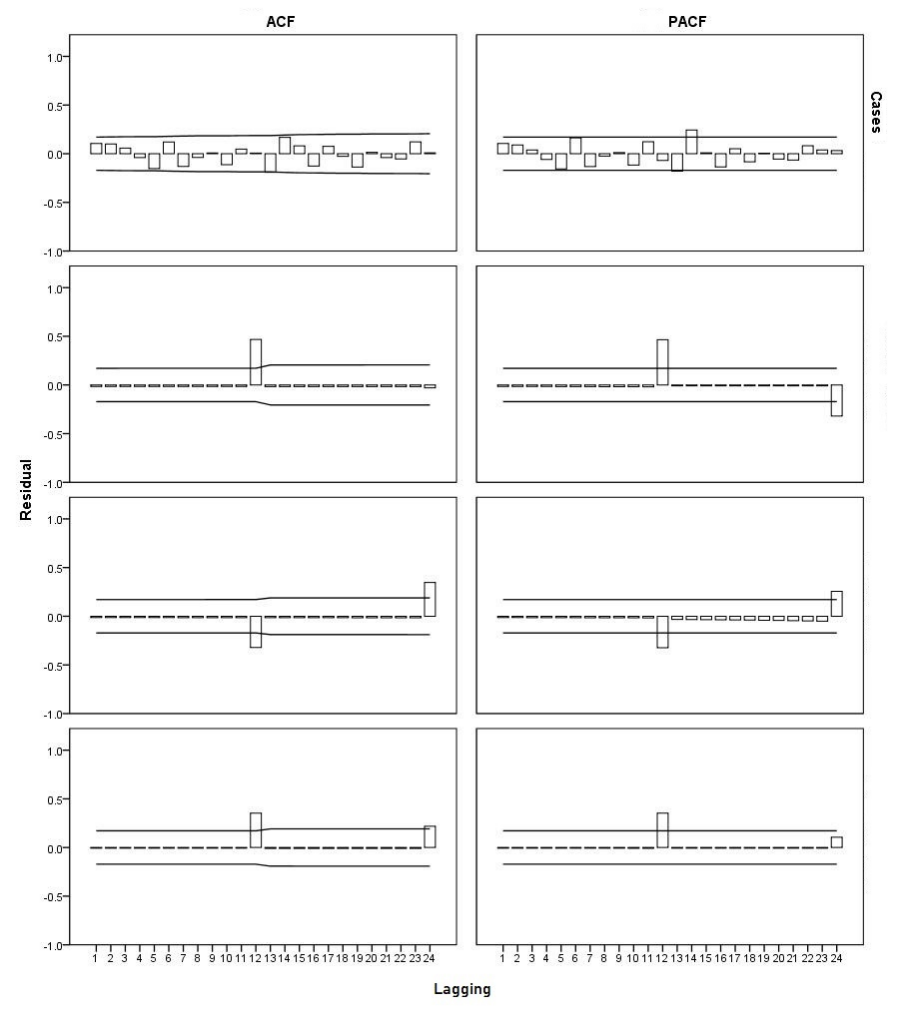
Transfer function-noise model: tuberculosis and incidence rate of ACF and PACF in the whole population. ACF: autocorrelation function; PACF: partial autocorrelation function.

The TFN model, number and incidence rate of TB cases, autocorrelation function, and partial autocorrelation function showed that the data sequence reached a stable state, and the effect of white noise was eliminated (Figure 5).

We found that the total illiteracy rate and average pressure at each measuring station (in individuals aged 15-24 years) were risk factors for TB incidence overall. Factors that protected against TB incidence were the number of families with the minimum living standard guarantee in urban areas, average wind speed, number of patients with invasive TB discharged, number of people with the minimum living standard guarantee in rural areas, total health expenditure as a percentage of regional GDP, being a single man (aged 0-14), number of hospitals, number of health workers in infectious disease hospitals (for those aged 25-64 years), amount of daily morning and evening exercises, number of people with the urban minimum living standard guarantee, and being a married woman (aged ≥65 years). The noise transfer-function model predicts that until 2035, the incidence of TB in male patients aged 0-14 and 15-24 years will increase, while in female patients aged 0-14 and ≥65 years, the incidence will increase rapidly.

## Discussion

### Principal Findings

To our knowledge, this is the most comprehensive ecological correlation and time series trend forecast analyses to investigate the associations of ecological factors with TB epidemic and the secular trend of TB incidence. The ecological correlation analyses demonstrated an overall limited influence of the environmental and socioeconomic effects and prediction on the TB epidemic. Incorporating these environmental and socioeconomic conditions into time series trend forecast models found that the TB incidence rate will continue to increase among male patients aged 0-24 years, female patients aged 0-14 years, and female patients older than 64 years, which should be considered as targeted populations to end the TB epidemic before 2035.

Compared with the worldwide rate and those for high-income countries, TB incidence in rural areas of Sichuan Province was very high, which was supported by a significant increase risk of TB infection among farmers compared with individuals with other occupations and in clustering areas of ethnic minorities in western and northeastern Sichuan [12]. Given that most people reside in rural areas worldwide, more effective strategies are needed to contain the TB epidemic.

Additionally, in China, the highest TB burden was found in those aged >70 years, the same as our findings that they had the peak TB incidence [22]; nevertheless, their incidence of this disease will not grow fast. Meanwhile, male patients aged 0-24 years, female patients aged 0-14 years, and female patients older than 64 years will have substantial growth by 2035 and take a heavy TB burden. Because of health problems and risk factors of chronic disease such as hypoimmunity, high systolic blood pressure, and high total cholesterol, with greater longevity, may predispose older adults to TB [22,23]. Apart from social aging and the above reasons, further national surveys to estimate the latent reasons for the increasing incidence of TB in female patients are required.

Of note, the risk factors for TB incidence varied across different ages. For children aged 0-14 years, we found that the proportion reflected by the total health expenditure divided by the regional GDP was associated with a decrease in the incidence of TB, while an inverse association was observed in the population older than 64 years. In-depth investigations need to reveal its role in TB incidence. In addition, a decrease in the number of forest fires led to a decrease in TB incidence. This finding was in line with those of Chen et al [11], who reported an association between decreased per capita living space and TB incidence. Forest fires may lead to air pollution, causing more patients with TB to seek diagnosis and treatment. Chen et al [11] suggested that future studies should consider social factors such as income and education. Furthermore, we found that in individuals aged 15-24 years, the total illiteracy rate can predict the incidence of TB.

For female individuals older than 64 years, daily morning and evening exercise, the number of people with the urban minimum living standard guarantee, and marriage (for women) may reduce the incidence of TB. Married women who do not require assistance for extreme poverty may have better nutrition and better health well-being [1,2], while the urban minimum living standard guarantees and the aid of a spouse provides support to seek TB treatment, especially for older persons (aged ≥65 years). Collectively, social and environmental supports also help control TB incidence in Sichuan.

Moreover, our findings did not support the protective role of neonatal BCG vaccination in TB incidence across all age groups. Notably, delivering the neonatal BCG vaccination displayed a negative correlation with TB incidence across all age groups, principally in line with previous findings [1,2]. Given that BCG vaccines offer the best chance to contain the accelerating spread of multidrug-resistant TB, more data are required to draw definitive conclusions on its role in TB incidence.

### Limitations

This study also has several limitations. First, we did not take into account determinations of genetic predisposition, treatments, treatment efficacy, and other ecological factors due to limited data availability. Second, all data in this study was obtained from Sichuan Province, and the results were subject to the inherent limitations of an ecological study; thus, principal findings may not be generalizable. Prospectively, further long-term cohort studies with large sample sizes as well as randomized controlled experimental studies are needed to determine the causal relationships.

### Conclusion

In conclusion, our study identifies a series of environmental and socioeconomic conditions associated with TB incidence through the most comprehensive ecological analyses. Incorporating these TB factors into the TFN model clarifies that male individuals aged 0-24 years, female individuals aged 0-14 years, and female individuals older than 64 years are barriers to the End TB Strategy in Sichuan, and provides new insights into TB prevention and control strategies.

## Appendix

Multimedia Appendix 1 Supplementary material.

## Abbreviations

ARIMA: autoregressive integrated moving average
DW: Durbin-Watson
GDP: gross domestic product
TB: tuberculosis
TFN: transfer function-noise

## Data Availability

All data used in the analysis of this manuscript are freely available by contacting the corresponding author. Data are available in a public open access repository, available on reasonable request.
