# Supplementary material for: The Environmental and Socioeconomic Effects and Prediction of Patients With Tuberculosis in Different Age Groups in Southwest China: A Population-Based Study
Source: JMIR Public Health Surveill. 2023 Jan 13;9:e40659. doi: 10.2196/40659 (PMC9883735; doi:10.2196/40659)
Supplement: Multimedia Appendix 1 [file publichealth_v9i1e40659_app1.doc]

*Supplementary materials*

**The Environmental and Socioeconomic Effects and Prediction of Tuberculosis Patients in different age groups in the Southwest of China**

Table S1 The correlation between the incidence rate of TB in different age groups and other diseases as well as health resources in Sichuan Province from 2006 to 2017

| Indicator | 0-14 years old | | | 15-24 years old | | | 25-64 years old | | | >64 years old | | |
| --- | --- | --- | --- | --- | --- | --- | --- | --- | --- | --- | --- | --- |
|  | male | female | total | male | female | total | male | female | total | male | female | total |
| coinfection with TB and HIV | -0.762** | -0.583* | -0.715** | -0.580* | -0.705* | -0.660* | -0.924** | -0.931** | -0.944** | -0.880** | -0.849** | -0.877** |
| existing HIV/AIDS | -0.794** | -0.605* | -0.744** | -0.614* | -0.744** | -0.688* | -0.948** | -0.949** | -0.955** | -0.896** | -0.862** | -0.893** |
| new HIV/AIDS case | -0.839** | -0.666* | -0.794** | -0.710** | -0.768** | -0.757** | -0.947** | -0.936** | -0.948** | -0.896** | -0.853** | -0.888** |
| HIV/AIDS deaths | -0.862** | -0.694* | -0.821** | -0.724** | -0.821** | -0.787** | -0.973** | -0.971** | -0.976** | -0.951** | -0.919** | -0.946** |
| respiratory disease mortality rate (1/100,000) | 0.715** | 0.567 | 0.680* | 0.525 | 0.755** | 0.619* | 0.856** | 0.835** | 0.838** | 0.804** | 0.762** | 0.803** |
| diabetes mortality rate (1/100,000) | -0.771** | -0.607* | -0.727** | -0.711** | -0.730** | -0.744** | -0.771** | -0.758** | -0.768** | -0.748** | -0.699* | -0.730** |
| total health expenditure as a percentage of GDP (%) | -0.945** | -0.824** | -0.921** | -0.855** | -0.900** | -0.903** | -0.941** | -0.933** | -0.943** | -0.939** | -0.908** | -0.935** |
| number of beds in hospitals per 1,000 people | -0.863** | -0.691* | -0.820** | -0.696* | -0.846** | -0.778** | -0.975** | -0.968** | -0.974** | -0.937** | -0.891** | -0.929** |
| health workers per 1,000 people | -0.850** | -0.673* | -0.805** | -0.684* | -0.831** | -0.764** | -0.967** | -0.959** | -0.967** | -0.927** | -0.879** | -0.919** |
| practicing physicians per 1,000 people | -0.938** | -0.781** | -0.902** | -0.795** | -0.924** | -0.865** | -0.990** | -0.975** | -0.980** | -0.970** | -0.921** | -0.962** |
| practicing (assistant) physicians per 1,000 people | -0.872** | -0.703* | -0.829** | -0.727** | -0.854** | -0.799** | -0.972** | -0.960** | -0.968** | -0.941** | -0.888** | -0.930** |
| registered nurses per 1,000 people | -0.874** | -0.696* | -0.828** | -0.732** | -0.840** | -0.796** | -0.969** | -0.956** | -0.965** | -0.930** | -0.874** | -0.918** |
| number of public health workers per 1,000 people | -0.687* | -0.447 | -0.618* | -0.502 | -0.712** | -0.574 | -0.810** | -0.752** | -0.764** | -0.727** | -0.626* | -0.708* |
| number of hospitals | -0.775** | -0.562 | -0.717** | -0.568 | -0.748** | -0.657* | -0.942** | -0.937** | -0.944** | -0.887** | -0.840** | -0.881** |
| primary medical and health institutions | -0.703* | -0.535 | -0.661* | -0.487 | -0.728** | -0.595* | -0.868** | -0.886** | -0.876** | -0.852** | -0.845** | -0.861** |
| funds for tuberculosis control (Chinese yuan; CNY) | 0.103 | 0.065 | 0.095 | -0.001 | 0.250 | 0.096 | 0.210 | 0.151 | 0.189 | 0.239 | 0.187 | 0.235 |
| number of infectious disease hospitals | -0.803** | -0.651* | -0.765** | -0.694* | -0.786** | -0.752** | -0.768** | -0.751** | -0.762** | -0.769** | -0.723** | -0.758** |
| number of beds in infectious disease hospitals | -0.811** | -0.630* | -0.763** | -0.612* | -0.726** | -0.685* | -0.920** | -0.925** | -0.934** | -0.855** | -0.812** | -0.849** |
| number of health workers in infectious disease hospitals | -0.789** | -0.625* | -0.747** | -0.604* | -0.739** | -0.692* | -0.890** | -0.907** | -0.915** | -0.857** | -0.829** | -0.853** |
| number of discharged patients (invasive TB) | -0.094 | -0.139 | -0.112 | 0.009 | 0.037 | 0.028 | -0.163 | -0.235 | -0.186 | -0.109 | -0.180 | -0.135 |
| average length of stay (invasive TB) | 0.714** | 0.441 | 0.634* | 0.505 | 0.714** | 0.593* | 0.865** | 0.828** | 0.844** | 0.772** | 0.683* | 0.755** |
| medical expenses per discharged patient (CNY)  (invasive pulmonary TB) | -0.244 | -0.169 | -0.224 | -0.087 | -0.091 | -0.107 | -0.356 | -0.444 | -0.409 | -0.275 | -0.340 | -0.298 |
| number of registered new smear positive pulmonary TB patients | 0.703* | 0.428 | 0.623* | 0.490 | 0.688* | 0.578* | 0.859** | 0.835** | 0.847** | 0.762** | 0.686* | 0.748** |
| number of cured patients | 0.704* | 0.429 | 0.623* | 0.490 | 0.687* | 0.577* | 0.860** | 0.836** | 0.848** | 0.763** | 0.687* | 0.748** |
| cure rate (%) | -0.461 | -0.215 | -0.384 | -0.383 | -0.529 | -0.439 | -0.524 | -0.478 | -0.492 | -0.486 | -0.404 | -0.462 |
| BCG vaccination rate (%) | -0.886** | -0.857** | -0.889** | -0.927** | -0.829** | -0.911** | -0.792** | -0.776** | -0.776** | -0.821** | -0.793** | -0.809** |

Note: ** indicates significant correlation at the level of 0.01 (two-tailed); * indicates significant correlation at the 0.05 level (two-tailed).

Table S2 The correlation between the incidence rate of TB in different age groups and economic and social factors in Sichuan Province from 2006 to 2017

| Indicator | 0-14 years old | | | 15-24 years old | | | 25-64 years old | | | >64 years old | | |
| --- | --- | --- | --- | --- | --- | --- | --- | --- | --- | --- | --- | --- |
|  | male | female | total | male | female | total | male | female | total | male | female | total |
| GDP (100 million Chinese yuan; CNY) | -0.875** | -0.697* | -0.830** | -0.719** | -0.848** | -0.791** | -0.985** | -0.975** | -0.982** | -0.958** | -0.913** | -0.950** |
| GDP per capita (CNY) | -0.912** | -0.750** | -0.873** | -0.763** | -0.903** | -0.838** | -0.988** | -0.974** | -0.979** | -0.968** | -0.918** | -0.958** |
| total income per capita in urban residents (CNY) | -0.887** | -0.720** | -0.846** | -0.722** | -0.862** | -0.798** | -0.983** | -0.978** | -0.981** | -0.947** | -0.904** | -0.940** |
| total expenditure per person in urban residents (CNY) | -0.895** | -0.742** | -0.859** | -0.742** | -0.854** | -0.808** | -0.979** | -0.979** | -0.979** | -0.939** | -0.904** | -0.934** |
| urban residents’ per capita expenditure on medical care (CNY) | -0.828** | -0.628* | -0.775** | -0.637* | -0.790** | -0.707* | -0.949** | -0.938** | -0.937** | -0.879** | -0.825** | -0.871** |
| urban residents' expenditure on transportation and communication (CNY) | -0.904** | -0.750** | -0.868** | -0.758** | -0.887** | -0.837** | -0.984** | -0.974** | -0.987** | -0.963** | -0.919** | -0.956** |
| urban residents’ expenditure on education and culture (CNY) | -0.852** | -0.667* | -0.805** | -0.673* | -0.841** | -0.769** | -0.956** | -0.936** | -0.960** | -0.924** | -0.862** | -0.913** |
| total income per capita in rural residents (CNY) | -0.844** | -0.661* | -0.797** | -0.656* | -0.805** | -0.734** | -0.963** | -0.962** | -0.963** | -0.909** | -0.867** | -0.904** |
| total expenditure per rural resident (CNY) | -0.784** | -0.591* | -0.732** | -0.584* | -0.729** | -0.652* | -0.921** | -0.930** | -0.920** | -0.847** | -0.813** | -0.844** |
| rural residents’ per capita expenditure on medical care (CNY) | -0.818** | -0.631* | -0.770** | -0.620* | -0.788** | -0.704* | -0.953** | -0.956** | -0.955** | -0.900** | -0.860** | -0.895** |
| rural residents' expenditure on transportation and communication (CNY) | -0.789** | -0.592* | -0.737** | -0.589* | -0.747** | -0.665* | -0.931** | -0.931** | -0.930** | -0.860** | -0.816** | -0.855** |
| rural residents’ expenditure on education and culture (CNY) | -0.754** | -0.544 | -0.697* | -0.530 | -0.707* | -0.608* | -0.905** | -0.915** | -0.904** | -0.825** | -0.791** | -0.824** |
| number of unemployed individuals (10,000 people) | -0.713** | -0.487 | -0.650* | -0.485 | -0.683* | -0.562 | -0.878** | -0.880** | -0.864** | -0.787** | -0.743** | -0.782** |
| unemployment rate (%) | 0.770** | 0.700* | 0.759** | 0.793** | 0.749** | 0.809** | 0.687* | 0.670* | 0.688* | 0.754** | 0.722** | 0.746** |
| rural employed persons (10,000 people) | 0.884** | 0.704* | 0.838** | 0.720** | 0.861** | 0.796** | 0.982** | 0.972** | 0.978** | 0.946** | 0.896** | 0.937** |
| civil medical assistance (times used) | 0.104 | -0.046 | 0.058 | -0.043 | 0.145 | 0.028 | 0.276 | 0.303 | 0.287 | 0.265 | 0.263 | 0.275 |
| urban residents with basic medical insurance (10,000 people) | -0.934** | -0.790** | -0.901** | -0.809** | -0.911** | -0.871** | -0.985** | -0.971** | -0.978** | -0.970** | -0.920** | -0.958** |
| rural residents with basic medical insurance (10,000 people) | -0.635* | -0.550 | -0.617* | -0.540 | -0.616* | -0.603* | -0.772** | -0.788** | -0.796** | -0.752** | -0.740** | -0.751** |
| number of families with the urban minimum living standard guarantee (households) | -0.211 | -0.347 | -0.258 | -0.431 | -0.250 | -0.354 | 0.066 | 0.118 | 0.112 | -0.047 | -0.025 | -0.030 |
| number of people with the urban minimum living standard guarantee (person) | 0.271 | 0.065 | 0.208 | 0.041 | 0.199 | 0.121 | 0.553 | 0.586* | 0.589* | 0.453 | 0.449 | 0.462 |
| urban subsistence allowance (10,000 CNY) | -0.977** | -0.918** | -0.973** | -0.932** | -0.965** | -0.973** | -0.927** | -0.901** | -0.916** | -0.957** | -0.915** | -0.946** |
| number of families with the rural minimum living standard guarantee (households) | -0.962** | -0.894** | -0.954** | -0.941** | -0.945** | -0.962** | -0.883** | -0.846** | -0.856** | -0.906** | -0.852** | -0.889** |
| number of people with the rural minimum living standard guarantee (person) | -0.847** | -0.873** | -0.868** | -0.923** | -0.837** | -0.907** | -0.669* | -0.627* | -0.637* | -0.728** | -0.688* | -0.710** |
| relief and support for rural people in extreme poverty | -0.559 | -0.396 | -0.518 | -0.300 | -0.625* | -0.429 | -0.736** | -0.730** | -0.730** | -0.681* | -0.655* | -0.689* |
| rural subsistence allowance (10,000 CNY) | -0.932** | -0.817** | -0.909** | -0.808** | -0.921** | -0.879** | -0.984** | -0.972** | -0.983** | -0.980** | -0.940** | -0.973** |

Note: ** indicates significant correlation at the level of 0.01 (two-tailed); * indicates significant correlation at the 0.05 level (two-tailed).

Table S3 The correlation between the incidence rate of TB in different age groups and pollutant, forestry and meteorology variables in Sichuan Province from 2006 to 2017

| Indicators | 0-14 years old | | | 15-24 years old | | | 25-64 years old | | | >64 years old | | |
| --- | --- | --- | --- | --- | --- | --- | --- | --- | --- | --- | --- | --- |
|  | male | female | total | male | female | total | male | female | total | male | female | total |
| total effluent discharge (tons) | -0.753** | -0.534 | -0.693* | -0.521 | -0.726** | -0.607* | -0.905** | -0.898** | -0.894** | -0.829** | -0.774** | -0.823** |
| COD emissions (tons) | -0.757** | -0.677* | -0.744** | -0.622* | -0.805** | -0.711** | -0.811** | -0.817** | -0.810** | -0.865** | -0.846** | -0.863** |
| ammonia nitrogen emission (tons) | -0.768** | -0.678* | -0.752** | -0.626* | -0.814** | -0.716** | -0.828** | -0.833** | -0.827** | -0.875** | -0.852** | -0.873** |
| SO_2_ emissions (tons) | 0.830** | 0.660* | 0.788** | 0.630* | 0.830** | 0.727** | 0.935** | 0.932** | 0.935** | 0.917** | 0.873** | 0.912** |
| NOx emissions (tons) | 0.688* | 0.452 | 0.621* | 0.442 | 0.614* | 0.522 | 0.843** | 0.840** | 0.845** | 0.730** | 0.678* | 0.725** |
| smoke dust emission (tons) | -0.618* | -0.516 | -0.596* | -0.448 | -0.566 | -0.481 | -0.718** | -0.758** | -0.707* | -0.667* | -0.691* | -0.682* |
| coal consumption (tons) | -0.059 | -0.219 | -0.111 | -0.335 | -0.118 | -0.240 | 0.202 | 0.253 | 0.250 | 0.082 | 0.097 | 0.100 |
| oil consumption (tons) | -0.883** | -0.702* | -0.837** | -0.706* | -0.855** | -0.783** | -0.980** | -0.968** | -0.974** | -0.935** | -0.883** | -0.927** |
| per capita parks and green area (m^2^) | -0.940** | -0.814** | -0.914** | -0.817** | -0.912** | -0.884** | -0.987** | -0.980** | -0.989** | -0.979** | -0.942** | -0.972** |
| forest area (10,000 Ha) | -0.945** | -0.858** | -0.930** | -0.947** | -0.898** | -0.954** | -0.895** | -0.874** | -0.882** | -0.917** | -0.871** | -0.901** |
| forest resource coverage rate (%) | -0.950** | -0.873** | -0.940** | -0.911** | -0.926** | -0.945** | -0.930** | -0.925** | -0.927** | -0.957** | -0.934** | -0.951** |
| number of forest fires | 0.430 | 0.471 | 0.450 | 0.359 | 0.255 | 0.354 | 0.400 | 0.423 | 0.439 | 0.335 | 0.347 | 0.338 |
| total area of forest fires (Ha) | 0.200 | -0.005 | 0.135 | -0.038 | 0.149 | 0.079 | 0.418 | 0.432 | 0.476 | 0.357 | 0.310 | 0.354 |
| total area affected by forest fires (Ha) | 0.025 | -0.129 | -0.025 | -0.202 | 0.039 | -0.104 | 0.221 | 0.238 | 0.242 | 0.171 | 0.149 | 0.174 |
| small-droplet evaporation | 0.190 | 0.209 | 0.195 | 0.377 | 0.125 | 0.278 | 0.060 | 0.051 | 0.039 | 0.076 | 0.067 | 0.058 |
| mean surface temperature | 0.196 | 0.175 | 0.187 | 0.356 | 0.155 | 0.259 | 0.067 | 0.054 | 0.026 | 0.087 | 0.081 | 0.070 |
| cumulative precipitation in 24 hours (20:00-20:00) | -0.595* | -0.434 | -0.551 | -0.389 | -0.486 | -0.456 | -0.557 | -0.550 | -0.576* | -0.448 | -0.386 | -0.438 |
| average pressure at each measuring station | 0.404 | 0.361 | 0.395 | 0.449 | 0.509 | 0.483 | 0.359 | 0.346 | 0.344 | 0.428 | 0.399 | 0.413 |
| mean relative humidity | -0.376 | -0.265 | -0.345 | -0.262 | -0.277 | -0.255 | -0.514 | -0.571 | -0.515 | -0.434 | -0.478 | -0.450 |
| daylight duration | 0.082 | -0.024 | 0.042 | 0.212 | -0.009 | 0.094 | 0.047 | 0.053 | 0.004 | 0.006 | -0.002 | -0.010 |
| average temperature | -0.122 | 0.015 | -0.081 | 0.033 | -0.113 | -0.035 | -0.281 | -0.273 | -0.296 | -0.192 | -0.164 | -0.198 |
| average wind speed | -0.418 | -0.256 | -0.372 | -0.268 | -0.420 | -0.270 | -0.445 | -0.448 | -0.377 | -0.357 | -0.341 | -0.360 |

Note: ** indicates significant correlation at the level of 0.01 (two-tailed); * indicates significant correlation at the 0.05 level (two-tailed).

Table S4 The correlation between the incidence rate of TB in different age groups and population structure, marriage and habit variables in Sichuan Province from 2006 to 2017

| Indicators | 0-14 years old | | | 15-24 years old | | | 25-64 years old | | | >64 years old | | |
| --- | --- | --- | --- | --- | --- | --- | --- | --- | --- | --- | --- | --- |
|  | male | female | total | male | female | total | male | female | total | male | female | total |
| urbanization rate (%) | -0.897** | -0.728** | -0.856** | -0.749** | -0.864** | -0.817** | -0.991** | -0.981** | -0.988** | -0.961** | -0.915** | -0.952** |
| population (persons) | -0.095 | -0.161 | -0.118 | -0.270 | -0.075 | -0.206 | 0.079 | 0.099 | 0.084 | 0.049 | 0.031 | 0.046 |
| sex ratio | 0.310 | 0.225 | 0.293 | -0.012 | 0.347 | 0.142 | 0.469 | 0.530 | 0.511 | 0.422 | 0.467 | 0.453 |
| population density (persons/km^2^) | -0.062 | 0.068 | -0.020 | 0.152 | 0.028 | 0.081 | -0.282 | -0.317 | -0.329 | -0.153 | -0.149 | -0.159 |
| life expectancy | -0.107 | 0.110 | -0.040 | 0.149 | -0.127 | 0.041 | -0.384 | -0.416 | -0.405 | -0.316 | -0.313 | -0.331 |
| male life expectancy | -0.050 | 0.158 | 0.015 | 0.197 | -0.066 | 0.102 | -0.310 | -0.347 | -0.327 | -0.237 | -0.244 | -0.255 |
| female life expectancy | -0.192 | 0.044 | -0.119 | 0.079 | -0.207 | -0.035 | -0.478 | -0.504 | -0.498 | -0.408 | -0.388 | -0.417 |
| total dependency ratio (%) | 0.457 | 0.582* | 0.504 | 0.604* | 0.398 | 0.569 | 0.199 | 0.188 | 0.219 | 0.286 | 0.287 | 0.276 |
| child dependency ratio (%) | 0.930** | 0.911** | 0.938** | 0.925** | 0.881** | 0.939** | 0.832** | 0.818** | 0.827** | 0.865** | 0.838** | 0.853** |
| elderly population dependency ratio (%) | -0.632* | -0.460 | -0.586* | -0.451 | -0.639* | -0.510 | -0.815** | -0.810** | -0.784** | -0.752** | -0.718** | -0.750** |
| 0-14-year-olds (%) | 0.953** | 0.910** | 0.953** | 0.919** | 0.909** | 0.942** | 0.890** | 0.877** | 0.882** | 0.911** | 0.883** | 0.901** |
| 15-64-year-olds (%) | -0.452 | -0.578* | -0.499 | -0.599* | -0.395 | -0.564 | -0.193 | -0.183 | -0.214 | -0.281 | -0.284 | -0.272 |
| >64-year-olds (%) | -0.722** | -0.561 | -0.681* | -0.554 | -0.721** | -0.611* | -0.876** | -0.870** | -0.848** | -0.824** | -0.788** | -0.820** |
| total unmarried (%) | -0.082 | 0.197 | 0.012 | 0.081 | -0.098 | 0.021 | -0.257 | -0.196 | -0.228 | -0.117 | -0.003 | -0.088 |
| unmarried men (%) | -0.732** | -0.570 | -0.685* | -0.749** | -0.684* | -0.738** | -0.689* | -0.594* | -0.643* | -0.658* | -0.519 | -0.613* |
| unmarried women (%) | 0.300 | 0.264 | 0.285 | 0.509 | 0.172 | 0.392 | 0.148 | 0.085 | 0.120 | 0.155 | 0.062 | 0.112 |
| total married (%) | 0.709** | 0.535 | 0.663* | 0.554 | 0.769** | 0.635* | 0.806** | 0.741** | 0.761** | 0.759** | 0.664* | 0.738** |
| married men (%) | 0.443 | 0.248 | 0.385 | 0.265 | 0.509 | 0.360 | 0.593* | 0.513 | 0.557 | 0.518 | 0.406 | 0.496 |
| married women (%) | 0.875** | 0.760** | 0.851** | 0.779** | 0.913** | 0.825** | 0.890** | .857** | 0.843** | 0.883** | 0.828** | 0.869** |
| total divorces (%) | -0.826** | -0.662* | -0.785** | -0.675* | -0.833** | -0.748** | -0.952** | -0.929** | -0.934** | -0.911** | -0.858** | -0.902** |
| divorced men (%) | -0.795** | -0.618* | -0.749** | -0.641* | -0.782** | -0.711** | -0.932** | -0.906** | -0.920** | -0.878** | -0.817** | -0.867** |
| divorced women (%) | -0.843** | -0.696* | -0.809** | -0.696* | -0.871** | -0.771** | -0.955** | -0.938** | -0.932** | -0.927** | -0.886** | -0.921** |
| total widowed (%) | 0.694* | 0.620* | 0.681* | 0.604* | 0.656* | 0.683* | 0.672* | 0.685* | 0.719** | 0.714** | 0.708** | 0.714** |
| widowers (%) | 0.822** | 0.734** | 0.806** | 0.734** | 0.784** | 0.800** | 0.818** | 0.826** | 0.845** | 0.845** | 0.837** | 0.843** |
| widows (%) | 0.532 | 0.460 | 0.518 | 0.428 | 0.482 | 0.512 | 0.506 | 0.524 | 0.567 | 0.540 | 0.532 | 0.542 |
| adult smoking rate (%) | 0.636* | 0.760** | 0.690* | 0.618* | 0.660* | 0.658* | 0.431 | 0.415 | 0.427 | 0.491 | 0.494 | 0.491 |
| exposure to second-hand smoke among nonsmokers (%) | -0.851** | -0.832** | -0.860** | -0.751** | -0.826** | -0.794** | -0.831** | -0.850** | -0.826** | -0.815** | -0.838** | -0.823** |
| regular exercise rate (%) | 0.652* | 0.763** | 0.701* | 0.642* | 0.634* | 0.645* | 0.514 | 0.532 | 0.504 | 0.523 | 0.573 | 0.535 |
| amount of daily morning and evening exercise | -0.896** | -0.828** | -0.889** | -0.820** | -0.917** | -0.883** | -0.888** | -0.885** | -0.885** | -0.945** | -0.926** | -0.943** |
| fitness monitoring compliance rate (%) | -0.819** | -0.820** | -0.834** | -0.865** | -0.796** | -0.858** | -0.722** | -0.719** | -0.713** | -0.783** | -0.787** | -0.786** |

Note: ** indicates significant correlation at the level of 0.01 (two-tailed); * indicates significant correlation at the 0.05 level (two-tailed).

Table S5 The correlation between the incidence rate of TB in different age groups and educational investment as well as education level in Sichuan Province from 2006 to 2017

| Indicator | 0-14 years old | | | 15-24 years old | | | 25-64 years old | | | >64 years old | | |
| --- | --- | --- | --- | --- | --- | --- | --- | --- | --- | --- | --- | --- |
|  | male | female | total | male | female | total | male | female | total | male | female | total |
| total illiteracy rate (%) | 0.826** | 0.795** | 0.827** | 0.926** | 0.822** | 0.908** | 0.696* | 0.652* | 0.670* | 0.766** | 0.727** | 0.751** |
| male illiteracy rate (%) | 0.846** | 0.768** | 0.832** | 0.896** | 0.856** | 0.887** | 0.802** | 0.759** | 0.760** | 0.862** | 0.812** | 0.843** |
| female illiteracy rate (%) | 0.894** | 0.832** | 0.887** | 0.918** | 0.892** | 0.932** | 0.818** | 0.779** | 0.796** | 0.879** | 0.825** | 0.860** |
| education funding (10,000 Chinese yuan) | -0.878** | -0.706* | -0.835** | -0.718** | -0.851** | -0.791** | -0.989** | -0.981** | -0.985** | -0.953** | -0.912** | -0.947** |
| number of schools (secondary school and above) | -0.325 | -0.340 | -0.339 | -0.342 | -0.413 | -0.379 | -0.259 | -0.266 | -0.259 | -0.364 | -0.391 | -0.376 |
| number of students in school (middle school and above) | -0.556 | -0.648* | -0.596* | -0.679* | -0.608* | -0.675* | -0.376 | -0.370 | -0.373 | -0.515 | -0.536 | -0.517 |
| number of teachers (secondary school and above) | -0.944** | -0.827** | -0.921** | -0.851** | -0.951** | -0.913** | -0.953** | -0.939** | -0.944** | -0.973** | -0.937** | -0.966** |
| enrollment number (secondary school and above) | -0.394 | -0.514 | -0.442 | -0.499 | -0.467 | -0.510 | -0.237 | -0.247 | -0.245 | -0.381 | -0.428 | -0.393 |
| number of graduates (secondary school and above) | -0.870** | -0.824** | -0.869** | -0.865** | -0.905** | -0.900** | -0.786** | -0.761** | -0.766** | -0.858** | -0.829** | -0.850** |
| total not attending school (%) | -0.082 | -0.278 | -0.154 | -0.066 | -0.130 | -0.105 | 0.005 | -0.036 | -0.023 | -0.012 | -0.119 | -0.048 |
| boys who have never attended school (%) | 0.208 | 0.115 | 0.180 | 0.057 | 0.231 | 0.116 | 0.354 | 0.358 | 0.344 | 0.349 | 0.314 | 0.340 |
| girls who have never attended school (%) | 0.858** | 0.778** | 0.843** | 0.861** | 0.862** | 0.882** | 0.807** | 0.768** | 0.783** | 0.866** | 0.806** | 0.846** |
| total elementary school students (%) | 0.939** | 0.840** | 0.921** | 0.883** | 0.917** | 0.929** | 0.919** | 0.914** | 0.922** | 0.944** | 0.918** | 0.937** |
| primary school boys (%) | 0.937** | 0.834** | 0.918** | 0.886** | 0.919** | 0.932** | 0.918** | 0.906** | 0.918** | 0.945** | 0.913** | 0.936** |
| primary school girls (%) | 0.933** | 0.839** | 0.917** | 0.869** | 0.906** | 0.916** | 0.914** | 0.916** | 0.919** | 0.932** | 0.916** | 0.929** |
| total junior high school students (%) | -0.468 | -0.563 | -0.505 | -0.617* | -0.455 | -0.568 | -0.330 | -0.345 | -0.325 | -0.430 | -0.442 | -0.419 |
| junior high school boys (%) | -0.422 | -0.470 | -0.442 | -0.494 | -0.412 | -0.463 | -0.356 | -0.383 | -0.346 | -0.430 | -0.442 | -0.421 |
| junior high school girls (%) | -0.479 | -0.463 | -0.479 | -0.575 | -0.388 | -0.536 | -0.498 | -0.499 | -0.515 | -0.545 | -0.554 | -0.540 |
| total high school students (%) | -0.884** | -0.689* | -0.833** | -0.756** | -0.865** | -0.814** | -0.966** | -0.943** | -0.949** | -0.950** | -0.893** | -0.937** |
| high school boys (%) | -0.845** | -0.644* | -0.791** | -0.692* | -0.827** | -0.754** | -0.962** | -0.943** | -0.942** | -0.928** | -0.870** | -0.916** |
| high school girls (%) | -0.901** | -0.725** | -0.856** | -0.798** | -0.888** | -0.858** | -0.957** | -0.935** | -0.949** | -0.956** | -0.905** | -0.945** |
| total college and upper level students (%) | -0.889** | -0.750** | -0.858** | -0.740** | -0.888** | -0.830** | -0.925** | -0.909** | -0.929** | -0.934** | -0.889** | -0.927** |
| college and upper level men (%) | -0.049 | -0.167 | -0.089 | -0.162 | 0.014 | -0.102 | 0.053 | 0.047 | 0.049 | 0.044 | 0.020 | 0.043 |
| college and upper level women (%) | -0.943** | -0.816** | -0.917** | -0.822** | -0.932** | -0.888** | -0.953** | -0.945** | -0.948** | -0.965** | -0.930** | -0.959** |

Note: ** indicates significant correlation at the level of 0.01 (two-tailed); * indicates significant correlation at the 0.05 level (two-tailed).

Table S6 Fitting of the tuberculosis transfer function-noise model on data from Sichuan Province during 2018-2035

| Model ID | Smooth R^2^ | R^2^ | Normalized BIC |
| --- | --- | --- | --- |
| individuals aged 0-14 | 0.536 | 0.983 | -3.507 |
| males aged 0-14 | 1.000 | 1.000 | -71.514 |
| females aged 0-14 | 0.279 | 0.870 | -2.307 |
| individuals aged 15-24 | 0.649 | 0.994 | -1.349 |
| males aged 15-24 | 0.998 | 1.000 | -5.080 |
| females aged 15-24 | 0.999 | 1.000 | -3.921 |
| females aged 25-64 | 1.000 | 1.000 | -50.644 |
| individuals aged >64 | 1.000 | 1.000 | -53.279 |
| males aged >64 | 1.000 | 1.000 | -48.402 |
| females aged >64 | 1.000 | 1.000 | -37.958 |
